# A Model for the Roll-Out of Comprehensive Adult Male Circumcision Services in African Low-Income Settings of High HIV Incidence: The ANRS 12126 Bophelo Pele Project

**DOI:** 10.1371/journal.pmed.1000309

**Published:** 2010-07-20

**Authors:** Pascale Lissouba, Dirk Taljaard, Dino Rech, Sean Doyle, Daniel Shabangu, Cynthia Nhlapo, Josephine Otchere-Darko, Thabo Mashigo, Caitlin Matson, David Lewis, Scott Billy, Bertran Auvert

**Affiliations:** 1 INSERM U1018 Centre de Recherche en Epidémiologie et Santé des Populations (CESP), Villejuif, France; 2 Progressus, Johannesburg, South Africa; 3 Society for Family Health, Johannesburg, South Africa; 4 National Institute for Communicable Diseases (NICD), National Health Laboratory Service (NHLS) Johannesburg, South Africa; 5 University of the Witwatersrand, Johannesburg, South Africa; 6 INSERM U1018 CESP, Hôpital Ambroise Paré, Assistance Publique-Hôpitaux de Paris, Boulogne, France; 7 University of Versailles-Saint-Quentin, Versailles, France; Rwanda Ministry of Health, Rwanda

## Abstract

**Background:**

World Health Organization (WHO)/Joint United Nations Programme on AIDS (UNAIDS) has recommended adult male circumcision (AMC) for the prevention of heterosexually acquired HIV infection in men from communities where HIV is hyperendemic and AMC prevalence is low. The objective of this study was to investigate the feasibility of the roll-out of medicalized AMC according to UNAIDS/WHO operational guidelines in a targeted African setting.

**Methods and Findings:**

The ANRS 12126 “Bophelo Pele” project was implemented in 2008 in the township of Orange Farm (South Africa). It became functional in 5 mo once local and ethical authorizations were obtained. Project activities involved community mobilization and outreach, as well as communication approaches aimed at both men and women incorporating broader HIV prevention strategies and promoting sexual health. Free medicalized AMC was offered to male residents aged 15 y and over at the project's main center, which had been designed for low-income settings. Through the establishment of an innovative surgical organization, up to 150 AMCs under local anesthesia, with sterilized circumcision disposable kits and electrocautery, could be performed per day by three task-sharing teams of one medical circumciser and five nurses. Community support for the project was high. As of November 2009, 14,011 men had been circumcised, averaging 740 per month in the past 12 mo, and 27.5% of project participants agreed to be tested for HIV. The rate of adverse events, none of which resulted in permanent damage or death, was 1.8%. Most of the men surveyed (92%) rated the services provided positively. An estimated 39.1% of adult uncircumcised male residents have undergone surgery and uptake is steadily increasing.

**Conclusion:**

This study demonstrates that a quality AMC roll-out adapted to African low-income settings is feasible and can be implemented quickly and safely according to international guidelines. The project can be a model for the scale-up of comprehensive AMC services, which could be tailored for other rural and urban communities of high HIV prevalence and low AMC rates in Eastern and Southern Africa.

*Please see later in the article for the Editors' Summary*

## Abstract 2

Bertrand Auvert and colleagues describe the large-scale roll-out of adult male circumcision through a program in South Africa.

## Editors' Summary

**Background:**

Acquired immunodeficiency syndrome (AIDS) has killed about 25 million people since 1981, and more than 30 million people (22 million in sub-Saharan Africa alone) are now infected with the human immunodeficiency virus (HIV), which causes AIDS. There is no cure for HIV/AIDS. Consequently, prevention of HIV infection is extremely important. Because HIV is most often spread through unprotected sex with an infected partner, individuals can reduce their risk of HIV infection by abstaining from sex, by having one or a few partners, and by always using a male or female condom. In addition, three trials in sub-Saharan Africa recently reported that medicalized adult male circumcision (AMC)—the surgical removal of the foreskin, a loose fold of skin that covers the head of the penis—can reduce HIV transmission rates in men by more than a half. Thus, AMC delivered as a catch-up campaign—in the long-term, circumcision of male infants is likely to be a more sustainable strategy—has the potential to reduce the prevalence of HIV (the proportion of the population infected with HIV) in sub-Saharan Africa.

**Why Was This Study Done?:**

The World Health Organization (WHO) and the Joint United Nations Programme on AIDS (UNAIDS) now recommend that AMC programs should be rolled-out wherever there is a generalized HIV epidemic and few men are circumcised. Accordingly, these organizations have defined a minimum package of AMC services and have issued guidelines and tools designed to engage communities in the roll-out and to ensure that appropriate AMC counseling and surgical facilities are available. But is rapid AMC roll-out feasible in real-life settings? Here, the researchers try to find out by studying the “Bophelo Pele” (Health First) project. This project, which follows the WHO/UNAIDS guidelines for AMC, aims to offer free, safe AMC services to all men aged 15 years or more living in the Orange Farm township in South Africa as part of a community-based intervention against HIV. Orange Farm is in a low-income region of South Africa where HIV prevalence is 15.2% and AMC prevalence is about 25%.

**What Did the Researchers Do and Find?:**

Before the Bophelo Pele project started in January 2008, the researchers consulted the community about the implementation of AMC, helped to create a community advisory board, organized community workshops to discuss the project, and surveyed people's knowledge about AMC and willingness to undergo AMC. These activities indicated a high level of community support for the project and a high level of willingness among men to undergo AMC. Once the project started, the researchers used multiple communication channels to tell the Orange Farm residents about AMC and broader HIV prevention strategies and provided eligible men with counseling about AMC and with voluntary HIV counseling and testing during the recruitment process. Three days after recruitment, eligible men were circumcised free-of-charge at the project's main center, where three teams of one medical circumciser and five nurses were able to complete up to 150 AMCs per day. By November 2009, 14,011 men had been circumcised (more than a third of the eligible men in the township), and AMC uptake was still increasing steadily. Nearly all the men circumcised over one 2-month period rated the AMC services positively in a survey and adverse effects (all mild) occurred after fewer than 1 in 50 circumcisions.

**What Do These Findings Mean?:**

These findings suggest that the rapid roll-out of high-quality, free AMC as an intervention against HIV has been successful in the Orange Farm township. However, other findings highlight some of the challenges that face AMC roll-out. For example, only a quarter of the participants agreed to voluntary HIV counseling and testing, which is worrying because newly circumcised HIV-positive men have an increased risk of transmitting HIV if they resume sexual activity too soon after the operation. Similarly, only two-thirds of the participants returned for a check-up after circumcision; this proportion needs to be increased to ensure the safety and efficacy of AMC programs. Nevertheless, these findings and those from similar intervention programs in Kenya and Uganda indicate that AMC scale-up should be feasible, at least in the short term, as an HIV prevention strategy in low-income communities where there is a high HIV prevalence and a low AMC rate.

**Additional Information:**

Please access these Web sites via the online version of this summary at http://dx.doi.org/10.1371/journal.pmed.1000309.

- Information is available from the US National Institute of Allergy and infectious diseases on HIV infection and AIDS
- HIV InSite has comprehensive information on all aspects of HIV/AIDS
- Information is available from Avert, an international AIDS charity on many aspects of HIV/AIDS, including information on HIV and AIDS in South Africa, and on circumcision and HIV (in English and Spanish)
- More information about male circumcision is available from WHO and from the Clearinghouse on Male Circumcision, including a June 2010 report from WHO/UNAIDS entitled Progress in male circumcision scale-up: country implementation and research update
- More information about the Bophelo Pele project is available

## Introduction

Because three randomized controlled trials conducted in sub-Saharan Africa have demonstrated a significant protective effect of about 60% of adult male circumcision (AMC) on male HIV acquisition [1]–[3], AMC has been recognized as an additional, important strategy for the prevention of heterosexually acquired HIV infection in men [4],[5]. Following a World Health Organization (WHO)/Joint United Nations Programme on AIDS (UNAIDS)-led international consultation, AMC scale-up was recommended to reduce the spread of HIV in countries where HIV is hyperendemic and AMC prevalence is low [5].

The potential public health benefits of this roll-out are substantial because AMC is expected to be both life-saving and cost-effective. A recent expert review has estimated that one HIV infection could be averted for every five to 15 AMCs performed [6]. Modeling studies have suggested that AMC generalization in sub-Saharan Africa could prevent 5.7 million new HIV infections and 3 million premature deaths among men, women, and children over a period of 20 y [7], and that in African countries with severe HIV epidemics, considerable reductions in HIV prevalence could be achieved over time among both men and women, ranging from 25% to 67% with AMC uptakes of 50% to 80% [8]. The cost savings from averted treatments are high, estimated at US$2.3 billion over 20 y [9], and, even with low coverage, AMC scale-up should generate large net savings after adjustment for averted HIV-related medical costs [10]. In addition, AMC has been shown to be protective against other sexually transmitted infections (STIs), such as herpes simplex virus-2 (HSV-2) [11],[12], human papillomavirus (HPV) [12],[13], and genital ulcer disease [14], and is associated with a reduction in the risk of genital cancers among both men and women [15],[16]. Furthermore, AMC, as part of a comprehensive HIV prevention package, is likely to have a complementary effect with other recommended strategies.

In view of these compelling arguments, and because there is evidence of high acceptability of the procedure in traditionally noncircumcising African communities [17], the rapid roll-out of AMC has become an ethical and public health imperative in countries with low AMC rates of Eastern and Southern Africa, the region where HIV prevalence is the highest in the world [18]. As indicated by modeling studies, AMC delivered as a catch-up campaign, aiming to increase AMC prevalence to about 80% within 5 y, would have an immediate impact on the HIV epidemic in these countries [7],[19]. To prepare this roll-out, WHO/UNAIDS has defined a minimum package of AMC services [20], and guidelines and tools to assist scale-up activities, such as community engagement and communication strategies [21], AMC counseling [22], and AMC surgical procedures [23] have been issued. However, little operational research has been generated so far with regards to the roll-out of safe, comprehensive, accessible, and acceptable services, and the feasibility of AMC scale-up in real-life circumstances according to these recommendations remains to be demonstrated.

The objective of this study was to test a generalizable model for providing optimized medicalized AMC services, and to investigate the feasibility of its rapid roll-out in a low-income South African community of high HIV prevalence and low AMC rates according to UNAIDS/WHO international recommendations and operational guidelines.

## Methods

### Ethics Statement

Ethical clearance was granted by the Human Research Ethics Committee (Medical) of the University of the Witwatersrand on May 8, 2007 (protocol study number M070367).

### ANRS 12126 Bophelo Pele Project General Presentation

The township of Orange Farm is located in the Gauteng province of South Africa, approximately 45 km from Johannesburg. It covers an area of about 50 km^2^ and has an estimated population of 200,000. The HIV epidemic in the province is among the most severe in the world, with prevalence estimated in 2009 at 15.2% among adults aged 15 to 49 y [24]. The first published randomized clinical trial of the effect of AMC on HIV acquisition was conducted in this community [1]. Orange Farm was considered a prime site in which to conduct operational research on AMC since it is a typical South African settlement where AMC is of low prevalence (22%–30%), but viewed positively [25].

The ANRS 12126 “Bophelo Pele” project, which translates as “Health First,” aims to offer free and safe AMC services to all men living in Orange Farm as a community-based intervention against HIV. The first AMC was performed in January 2008, 5 mo after funding had been made available and once local and ethical authorizations were obtained. The preparation of the project and its activities are detailed in the following methods sections. These activities involved community mobilization and communication strategies aimed at both men and women, which were delivered during outreach and at recruitment points located in Orange Farm. Following recruitment, consenting volunteers underwent AMC at the project's main center located in a highly visible building centrally located across a taxi rank and the Orange Farm train station. All project activities were monitored and evaluated throughout service delivery. For the running of the project, about 70 people were employed, including a site manager, a coordinator, two secretaries, 20 outreach field workers, 14 counselors, two nurses for voluntary counseling and testing (VCT), two lab technicians, four interviewers, two data entry clerks, three medical circumcisers, who were registered medical doctors as per South African Law, and 17 nurses.

### Preparation of the Project

Before the project's implementation, discussions with the Gauteng provincial government were conducted in order to obtain authorization and institutional support. Subsequently, qualitative and quantitative research activities, described below, were carried out to assist project preparation.

#### Community consultation, community advisory board, and community workshops

A community consultation was conducted in Orange Farm in December 2007 to query the Orange Farm community on the implementation of AMC in the township and assess the Bophelo project's acceptability. This consultation was led by an independent consultancy firm among Orange Farm residents, community leaders, and representatives of local organizations. Recommendations were made to proceed with the project's implementation and a community advisory board (CAB) was created, which was composed of local and regional political representatives, church leaders, medical doctors, primary health care representatives, representatives of various nongovernmental organizations (NGOs), and activists. All the project's documents and forms, including those pertaining to the communication, outreach, and counseling activities, were reviewed by the CAB. Concurrently, a series of baseline community workshops were organized by the project's investigators to present and discuss the Bophelo Pele project with community members, youth groups, local key partners, and stakeholders. Discussions were also initiated with local traditional circumcisers to plan collaborations.

#### Cross-sectional survey, qualitative studies, and counseling message assessment

To assess the project's acceptability quantitatively and provide baseline data on knowledge about AMC and willingness to undergo AMC among the Orange Farm community, a cross-sectional survey was conducted at baseline in 2007. The methodology used was that of a research study conducted in the same community in 2002 [26]. Furthermore, quality of AMC messaging and information recall were evaluated by a qualitative focus group discussion study conducted among six groups, two composed of women, two of uncircumcised men, and two of circumcised men. These participants were given gender-specific pamphlets about AMC in their preferred language, and were asked to read them and return after 4 mo to comment on them and participate in discussions on AMC. Finally, the effectiveness of the counseling messages was assessed through a nested study, in which a random sample of male volunteers were counseled, circumcised, and asked to return for a 2-mo follow-up visit. Data on counseling recall and adherence to behavioral directives were collected and analyzed.

### Communication Strategies and Outreach

In order to inform Orange Farm residents about the project, combined communication approaches, incorporating broader HIV prevention strategies, were employed at the individual, family, peer, and community levels. These approaches targeted both primary and secondary audiences and used multiple communication channels and social marketing tools. Information about the project was provided through weekly broadcasts on the local radio station, a mobile speaker system advertising once a week throughout the community, the use of a singing group during special events, and the organization of community block parties. Visits to all 53,000 Orange Farm households were performed door-to-door by outreach fieldworkers to provide interpersonal communication, and a second round of household visits was ongoing in November 2009. Oral and written information was delivered to men and women during home visits and at specific locations, including outside schools, at taxi ranks, clinics, shopping facilities, markets, and the train station. School talks were organized to give information about AMC and general HIV prevention education, and local clinics were visited by members of the project's medical team every day during 3-mo periods to promote AMC and inform patients and clinic staff of the services provided by the project. To enhance project recognition, the Bophelo Pele project logo was displayed on the facade of the project's main center, and appeared on all project-related printed documentation and items of clothing worn by members of the outreach team.

Information dissemination was achieved through the distribution of pamphlets published in local languages. These pamphlets provided details about the Bophelo Pele project and gave gender-specific information about AMC. The key message was that the project offered men aged 15 y and over free AMC performed by trained medical circumcisers. The flyers also explained the effect of AMC on HIV acquisition and transmission, stressing the facts that (a) AMC protection was only partial, (b) there were no direct benefits to women, (c) AMC did not replace consistent condom use, which was still required and strongly recommended, and (d) to allow for complete wound healing, a 6-wk abstinence period post-AMC needed to be respected before resuming sexual activity. Drawings of circumcised and uncircumcised penises were presented to volunteers to ensure that they correctly understood that the medicalized AMC procedure consisted of the complete removal of the foreskin.

### Recruitment

Two fixed and two mobile recruitment points were available throughout the community. The mobile recruitment points moved to a different area of Orange Farm every 3–4 wk.

Men aged 15 y and over were actively recruited door-to-door, from specific locations or popular gathering places, as described above, or referred to the project by local health clinics. These volunteers, in addition to all eligible men willing to be circumcised and coming of their own volition, were welcome at all recruitment points. Concurrently, some young men were recruited through collaboration with traditional circumcisers, whereby they received traditional initiation along with medicalized AMC services.

The recruitment process lasted about 1 h. It was composed of a group information session and individual counseling. At each recruitment point, group information sessions, individual counseling, and VCT were conducted separately. The group information session, offered to groups of up to 15 participants, was open to all community members, including parents, friends, and partners of young men. The goal of this session was to deliver information about AMC as well as public health messages on HIV prevention, such as the promotion of consistent condom use, the reduction of the number of sexual partners, HIV testing and risk reduction counseling (VCT), and STI treatment. A significant part of this session was devoted to explaining the risks and benefits of AMC and its association with HIV to ensure that the preventive role of AMC, in comparison with the role of other prevention strategies, was well understood. Individual counseling was subsequently offered to willing male and female session participants, either individually or as couples. During this counseling, recommendations on HIV prevention methods and sexual health were reinforced, safe sex messaging was adapted to the personal situation of the participant(s), and rapid HIV testing using two parallel tests was offered and strongly recommended.

AMC was offered to all volunteers, regardless of their HIV status. Volunteers who tested HIV-positive during VCT were offered a CD4 count and obtained their results in 20 min. If their CD4 was above 200, they could be circumcised by the project, but were strongly advised to join a local NGO or a local public or private health care facility that could provide care and support to HIV-positive people. Individuals with a CD4 count below 200 were first referred to one of these organizations to receive antiretroviral treatment as well as drugs to prevent opportunistic infections before undergoing AMC. In such cases, the project staff ensured that these men had initiated and received proper care.

To be circumcised by the project, proof of age was required, such as a National Identity Document or a birth certificate. Once volunteers had attended both information session and individual counseling, they were required to sign a consent form. Those aged 15 through 17 y were required to sign an assent form and present a signed parental consent form to be included. In addition, men aged 15 y needed to present a medical doctor's certificate stating that they would benefit from AMC for health reasons. These certificates were provided by the study medical circumcisers.

All volunteers received a personalized participant's card with their photo and were given a day appointment for AMC surgery at least 3 d after the recruitment process and no later than 10 d after. This 3-d period was designed to allow volunteers to have additional time to make their final decision before becoming project participants and undergoing AMC surgery.

### Surgery

Most AMCs were performed at the project's main center, which was placed under the auspices of the Department of Urology of the University of the Witwatersrand. This department provided AMC training for the project's medical circumcisers and nurses. For AMC surgery and the follow-up visit, participants were transported to and from the project's main center to the locations where they had been recruited.

On the day of their appointment and prior to AMC surgery, volunteers were asked to answer a short general health questionnaire and undergo a genital examination to check for the absence of AMC contraindications such as some penile abnormalities and signs of STI. In the event of symptomatic STI, the AMC surgery was rescheduled and participants were offered immediate treatment, either at the project's main center or at a local clinic, free of charge. Information collected with the health questionnaire and during the genital examination was recorded in a confidential participant's medical file.

The surgery room, adapted from that of a local circumcising medical doctor to optimize the use of space, was divided into 11 surgical bays. AMC surgeries were performed according to international recommendations [23], using the forceps guided method, electrocauterization for hemostasis (electrocautery), and sterilized disposable circumcision kits, in order to ensure practicality and maximize quality and speed. The circumcision kits used had been upgraded from a previous disposable kit designed by the project's research team [1]. Metallic surgical instruments, such as forceps, were recycled by the company producing the circumcision kits. The AMCs were standardized and performed using task-sharing by a medical team composed of five nurses and a medical circumciser. The nurses administered local anesthesia, assisted during surgery and completed suturing under the supervision of the medical circumciser. The medical circumciser applied the forceps, cut the foreskin, controlled the bleeding using electrocautery, initiated the suturing, and conducted a final check-up before participants were discharged. This surgical organization was specifically designed to handle a high number of AMCs. It was streamlined to optimize the medical circumciser's time spent per participant and control individual costs.

AMC surgery for traditional initiates was conducted in groups, according to the same method, but could only be performed by male medical staff and in the presence of the traditional circumciser, as per his request.

Some local medical doctors, who were already performing AMC in the community, were associated with the project and were referred participants for surgery. The surgical method and kits used were identical to the ones employed at the project's main center.

After the procedure and before leaving the center, participants were (a) given analgesics for the relief of pain to be taken within the next 2 d, (b) reminded by the nurses of the required 6-wk abstinence from sexual activities, including masturbation, and (c) given orally detailed postoperative instructions on wound care and management, follow-up visit attendance, and actions to take in case of an adverse event. These instructions were also included in a “postoperative care” written document provided in their preferred language.

Participants were asked to return for one follow-up visit, 2–4 d after AMC surgery, which was conducted at the project's main center by trained nurses. At least two reminder phone calls were placed to participants who had not returned for their follow-up visit. If they still would not attend, they received a follow-up consultation over the phone conducted by a member of the study staff.

In case of an adverse event, participants were advised to return to the project's main center immediately, or call the 24-h emergency toll-free phone number to reach the response team who could make home visits after hours. Once at the project's main center, they were referred by the attending nurse to one of the study's medical circumcisers. For adverse event follow-up, participants were collected at home and brought to the project's main center. They were monitored daily or weekly, depending on the type and severity of the adverse event.

### Project Monitoring and Evaluation

In order to provide feedback and incorporate the views of stakeholders and project beneficiaries, community involvement was encouraged. Community workshops were regularly organized, during which questions and concerns from stakeholders and community members were addressed to the project team. CAB meetings were held every 3 mo from the beginning of the project. To monitor the project's acceptability among the community, an evaluation study based on 11 focus group discussions among a total of 65 circumcised men, uncircumcised men, and female partners of both circumcised and uncircumcised men aged 15 to 49 y was conducted by independent researchers 15 mo after the beginning of the project. Finally, in August and September 2009, a postsurgery, anonymous, self-administered questionnaire was systematically proposed to all participants coming for AMC. The aim of this questionnaire was to collect information on participants' experiences and perceptions of the project. They were asked about (a) the communication source that had influenced them to join to the project, (b) their reason for undergoing AMC, (c) their opinion on the quality of the information provided during the information session and individual counseling, (d) their opinion on the on-site wait time for counseling and AMC surgery, (e) the likelihood that they would adhere to the 6-wk postsurgery abstinence period, and (f) their opinion on the quality of the services provided by the medical circumcisers. This questionnaire was completed by 1,158 participants, corresponding to a response rate of 85.8%.

Standard operating procedures were established for the recruitment, enrolment, counseling, and general processing of volunteers, the preparation of medical staff and participants, as well as the maintenance of the surgery room. These documents were regularly discussed and reviewed. In addition, independent audits of all the documents used for the outreach, communication, and recruitment activities, as well as all the forms used at the project's main center for surgery and follow-up, were conducted recurrently. External and internal quality controls using the WHO Quality Assessment Toolkit [27] were conducted.

The project's main center surgery was visited twice by the WHO Quality Assurance coordination team and was regularly audited by senior surgeons of the University of the Witwatersrand Urology Department. Independent audits on Good Clinical Practice were conducted every year. Collaborating medical doctors were regularly monitored by the project's medical staff, and feedback sessions were encouraged. Protocols for the identification and treatment of adverse events were established and adverse events management training was provided to medical circumcisers and nurses. All adverse events were described in detail, recorded in a specific adverse event form, and reported to the investigators and the Data and Safety Monitoring Board.

In addition to the confidential participant's medical file, as described above, a participant form was created in order to record basic demographic details on volunteers such as age and preferred language—Sesotho, IsiZulu, Setswana, or IsiXhosa—which was used as a proxy for ethnic distribution because there is frequent mixing between the Sothos, Zulus, Tswana, and Xhosa ethnic groups in the Orange Farm community. The dates of every contact with project services, including the information session, individual counseling, HIV testing, AMC surgery, and follow-up visit were also recorded on this form to allow for the monitoring of the project's activities and keep track of participant flow through the procedures.

## Results

Findings from the activities conducted before the start of the project were as follows: The community consultation showed high levels of acceptability and support for the project and the cross-sectional survey indicated that willingness to undergo AMC was high and predicted a substantial AMC uptake among uncircumcised adult men, who were for the most part unaware of the AMC trial that had been conducted previously in the community (Table 1). The focus group discussion study showed that the quality of AMC messaging was satisfactory and that information recall remained high in the 4-mo time frame (see Table S1). The nested study suggested that the vast majority of respondents displayed high counseling recall and reported high adherence to behavioral directives after 2 mo (Table 2).

**Table 1:** AMC knowledge and willingness to undergo AMC among a representative sample of Orange Farm male residents (*n* = 802).

Category	Percent Result (*n*)
***Background characteristics***	
**Age (y)**	
Under 21	52.2 (419)
21 and over	47.8 (383)
**Preferred language**	
Sesotho or Setswana	42.5 (341)
IsiZulu or IsiXhosa	55.2 (443)
Other	2.3 (18)
**Relationship status**	
Ever married or living with someone as married	6.5 (52)
Committed to someone but not living together	42.8 (343)
Single	50.7 (407)
**Secondary school level completed**	
Yes	26.4 (212)
No	73.6 (590)
**Self-reported circumcision status**	
Circumcised	30.4 (244)
Uncircumcised	69.6 (558)
***Knowledge of AMC trial conducted in Orange Farm in 2002–2005***	
**Know about AMC trial**	
Yes	36.0 (289)
No	64.0 (513)
**Know results of AMC trial**	
AMC reduces the risk of HIV infection	2.1 (17)
AMC stops the risk of HIV infection	0.4 (3)
Other answer	1.0 (8)
Do not know	96.5 (774)
***Knowledge about AMC and HIV risk***	
**HIV risk of self-reported circumcised men compared with uncircumcised men**	
Fully protected against HIV	9.4 (75)
Less at risk of being infected with HIV	51.6 (414)
Same risk of being infected with HIV	29.8 (239)
More at risk of being infected with HIV	9.2 (74)
**HIV risk of female partners of circumcised men**	
Fully or partially protected against HIV	5.2 (41)
Not protected against HIV	60.3 (484)
Do not know	34.5 (277)
***Willingness to undergo AMC***	
**If offered free of charge at doctor's office** a	
Would agree to get circumcised	74.6 (416)
Would not agree to get circumcised	25.4 (142)

Data are from the cross-sectional survey conducted in 2007.

a Among self-reported uncircumcised men.

**Table 2:** Counseling recall and adherence to behavioral directives among a subsample of men (*n* = 311) undergoing AMC.

Category	Percent Result (*n*)
***Background characteristics***	
**Age (y)**	
Under 21	48.2 (150)
21 and over	51.8 (161)
**Preferred language**	
Sesotho or Setswana	39.9 (124)
IsiZulu or IsiXhosa	59.8 (186)
Other	0.3 (1)
**Relationship status**	
Ever married or living with someone as married	3.9 (12)
Committed to someone but not living together	37.9 (118)
Single	58.2 (181)
**Secondary school level completed**	
Yes	32.2 (100)
No	67.8 (211)
***Recall of counseling messages 2 mo after counseling provision***	
**How long is the abstinence period after AMC?**	
6 wk	98.4 (306)
Other answer	1.6 (5)
**Can men have sexual intercourse during the six wk following AMC?**	
Yes	6.8 (21)
No	93.2 (290)
**What is the effect of AMC on HIV?**	
Reduction of the risk of infection in men	97.4 (303)
Other answer	2.6 (8)
**When circumcised, can men get infected with HIV?**	
Yes	99.0 (308
No	1.0 (3)
**Can risky sexual behavior counteract the benefits of AMC?**	
Yes	95.8 (298)
No	4.2 (13)
**Does AMC protect women from getting infected with HIV?**	
Yes	16.4 (51)
No	83.6 (260)
**Once circumcised, is it still necessary to use condoms?**	
Yes	99.4 (309)
No	0.6 (2)
***Adherence to behavioral directives***	
**Had sexual intercourse in the 6 wk following AMC?**	
Yes	12.5 (39)
No	87.5 (272)
**Protected sexual intercourse in the 6 wk following AMC** a **?**	
Yes	82.1 (32)
No	17.9 (7)

a Among those having had sexual intercourse in the 6 wk following AMC.

From the community workshops conducted during the course of the study, it appeared that the Bophelo Pele project was well known within the community, that it was receiving good public support, and that its goals were well perceived. Feedback from CAB meetings, which were held as planned, indicated that the issues most frequently raised were the dissemination of the program to other neighboring townships, where demand for AMC services was increasing, and the community's wish for the enrollment of younger adolescents. Results from the evaluation study showed that all participants rated the project's reliability, the usefulness of the information provided, and staff friendliness as excellent or good. The project's accessibility and turnaround time were rated as excellent or good by 93.3% of participants.

The project's main center surgery was satisfactorily audited by the WHO Quality Assurance coordination team and by officials of the University of the Witwatersrand, and favorably reviewed through independent Good Clinical Practice audits. With the surgical team composition and the task-sharing set-up, six to ten AMCs could be performed per hour, using four surgical bays, for an average medical circumciser time of 7.5 min per AMC, and a total procedure time of 20 min. The surgery had the capacity to perform up to 150 AMCs per day. With this optimized use of space and personnel, AMC cost was reduced to ZAR300 per AMC (about EUR30 or US$40). This estimate consisted of the salaries of the surgical team, as well as the cost of the circumcision kits and of the rental and maintenance of the surgical space.

**Figure 1:**
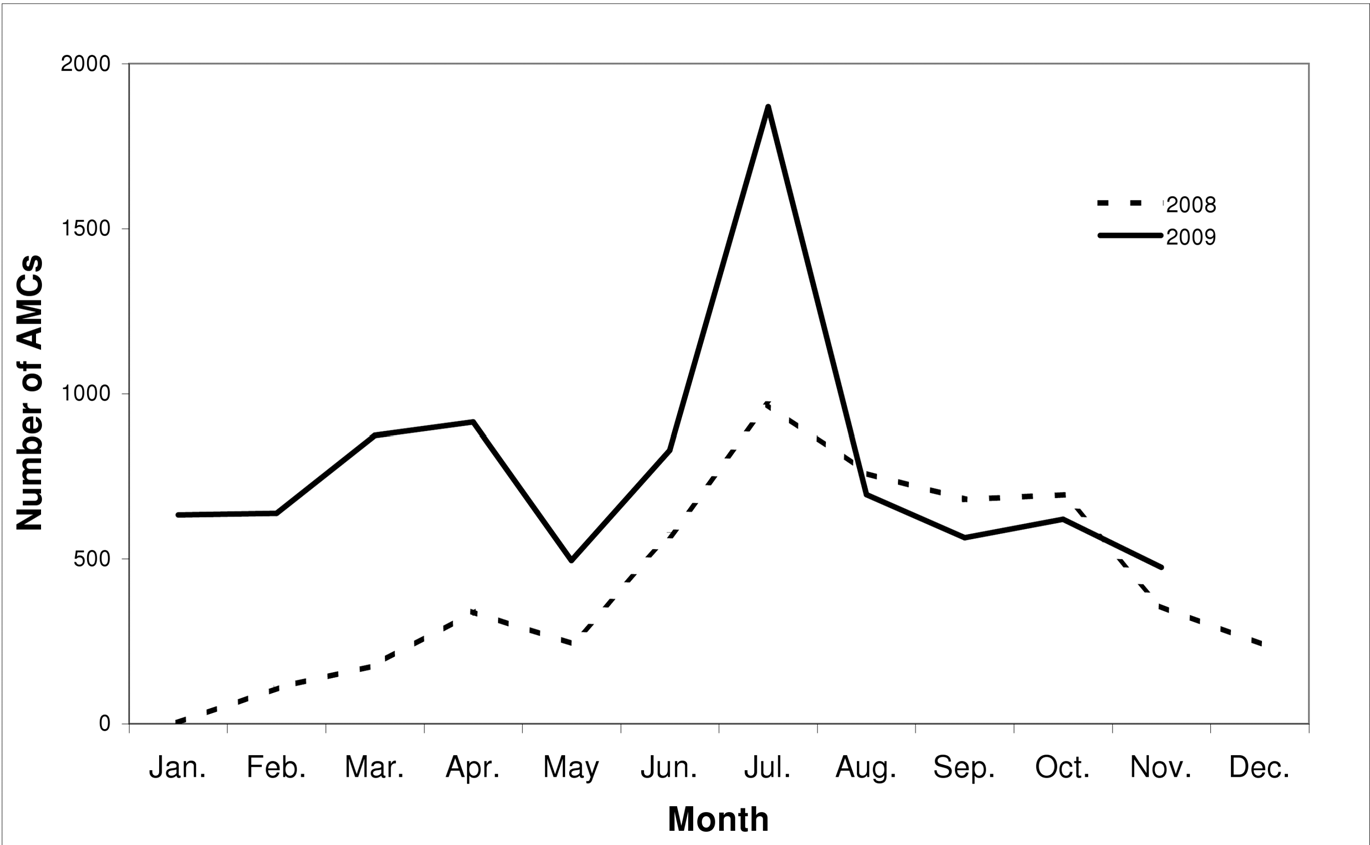
Number of AMCs performed monthly through the project in 2008 and 2009.

As of November 2009, a total of 14,011 AMCs had been performed, averaging 740 per month in the preceding year. Most AMC surgeries occurred at the project's main center, but 265 AMCs (1.9%) were carried out by the three Orange Farm collaborating medical doctors. About two-thirds (67%) of participants returned for their follow-up visit. From the initial survey, it was estimated that 35,851 clinically uncircumcised men, aged 15 y and over, lived in Orange Farm. AMC uptake, defined as the proportion of men undergoing the procedure among all eligible men in the township, was calculated to be 39.1% (14,011/35,851). Figure 1 shows the number of AMCs performed per month since the beginning of the project. It appears that the saturation level, which is the point when all willing eligible men have been circumcised, has not yet been reached and that uptake is still increasing. This observation is suggested by the facts that (a) the number of AMCs performed in the last months of 2009 was comparable to the number performed during the corresponding months in 2008 (Figure 1); (b) residents who had moved to Orange Farm in the year before being circumcised represented only 13% of the AMCs performed in the previous 2 mo; and (c) assuming a birth rate of 18/1,000, the number of AMCs needed to circumcise all young men turning 15 every month was estimated to be less than 150, which was only about a fifth of the number of AMCs performed monthly in the prior year.

The rate of adverse events since the beginning of the project was 1.8% (257/14,011). Most were due to bleeding, infections, and swelling. Thirteen adverse events occurred during surgery. Among all adverse events, nine resulted in participant hospitalization, representing 0.064% of the AMCs performed. They were all treated without any major permanent damage. However, one case resulted in minor scarring on the penis.

A total of 4,760 VCT sessions were performed, 901 among women, and 3,859 among men, which corresponded to 27.5% of those who underwent AMC. HIV prevalence among female and male participants was 18.9% and 8.7%, respectively.

**Figure 2:**
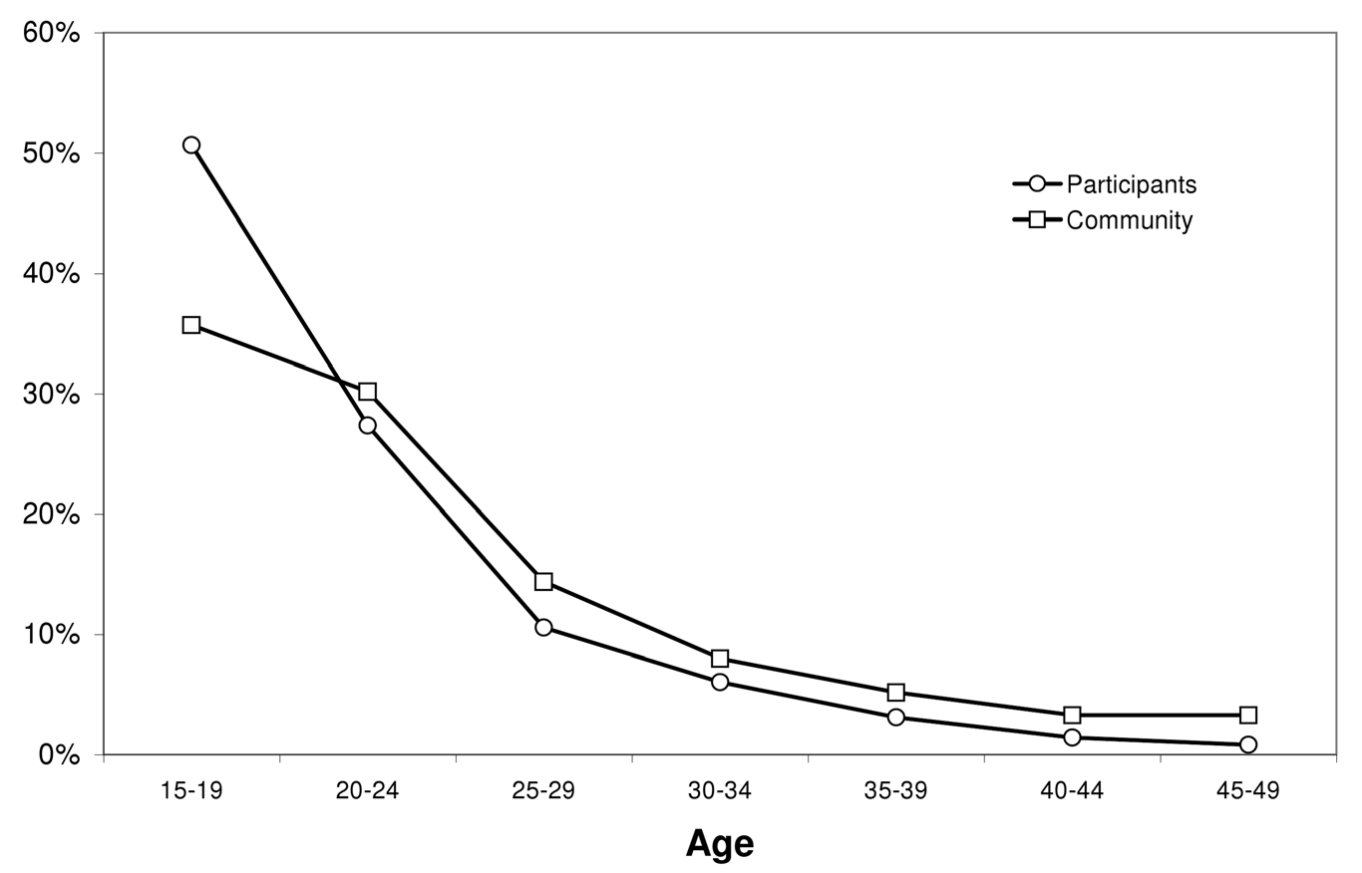
Age distribution among project participants and men of the community.

Figure 2 illustrates the age distribution of the project's participants and men in the community in the 15- to 49-y age range, using data collected from both the cross-sectional survey and the participant's form. Among participants, the mean (median) age in years was 21.6 (19). Although the oldest volunteer was 68 y old, participants were on average younger than men from the broader Orange Farm community, among whom mean (median) age was estimated at 24.5 y (22). As shown in Table 3, there is a slightly lower proportion of men speaking IsiZulu among project's participants in comparison with their proportion in the population, which is probably due to the fact that Zulus do not traditionally practice circumcision. The first group of 11 Sotho traditional initiates was brought in by a traditional circumciser in November 2009 and more collaboration is expected in the future.

**Table 3:** Language distribution among project participants and men of the community.

Language	Percent Participants (*n* = 14,011)	Percent Community (*n* = 802)
**Sesotho**	49.0	40.8
**IsiZulu**	42.2	54.5
**Other**	8.8	4.7

**Figure 3:**
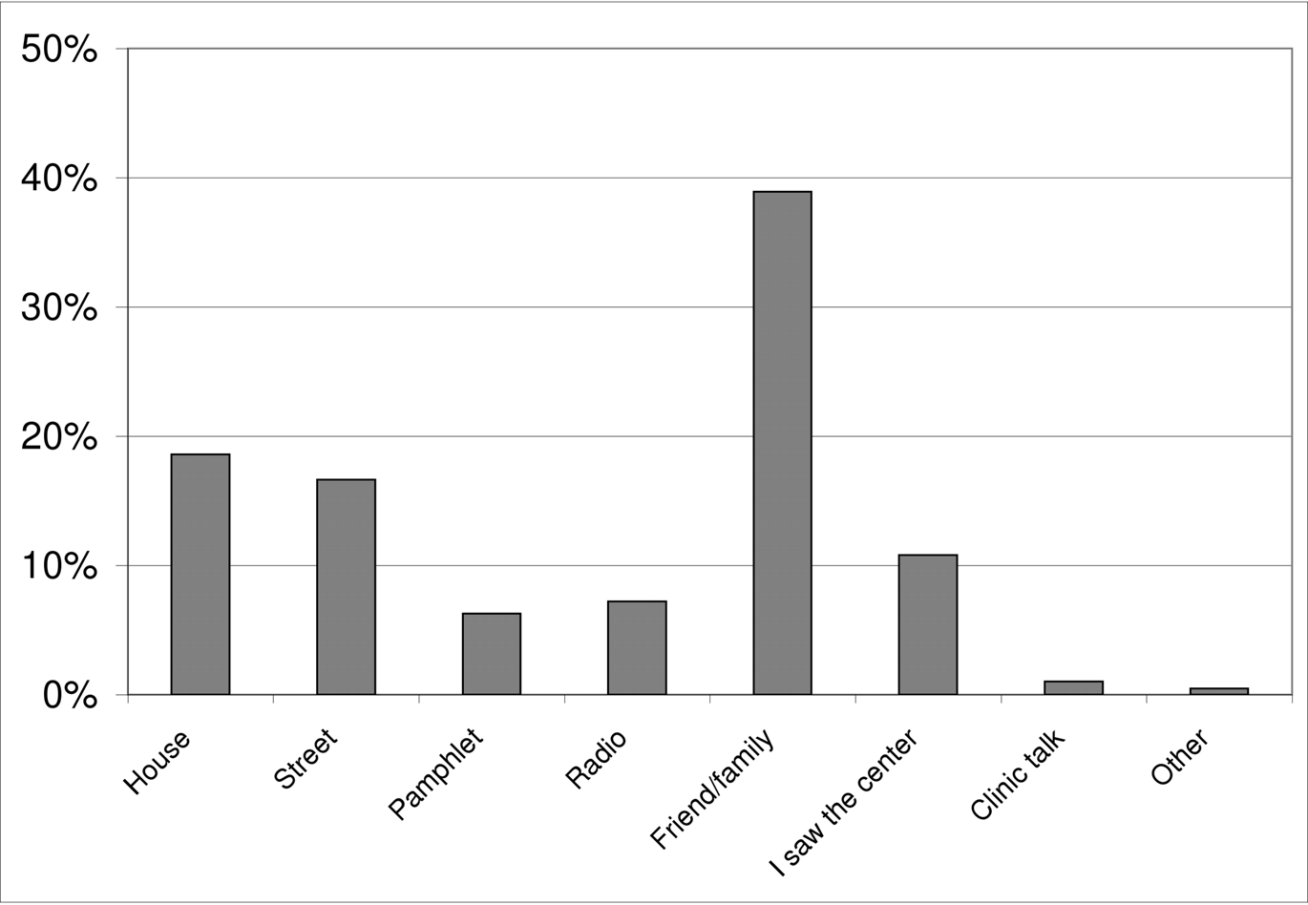
Communication sources that influenced participants to join the project.

**Figure 4:**
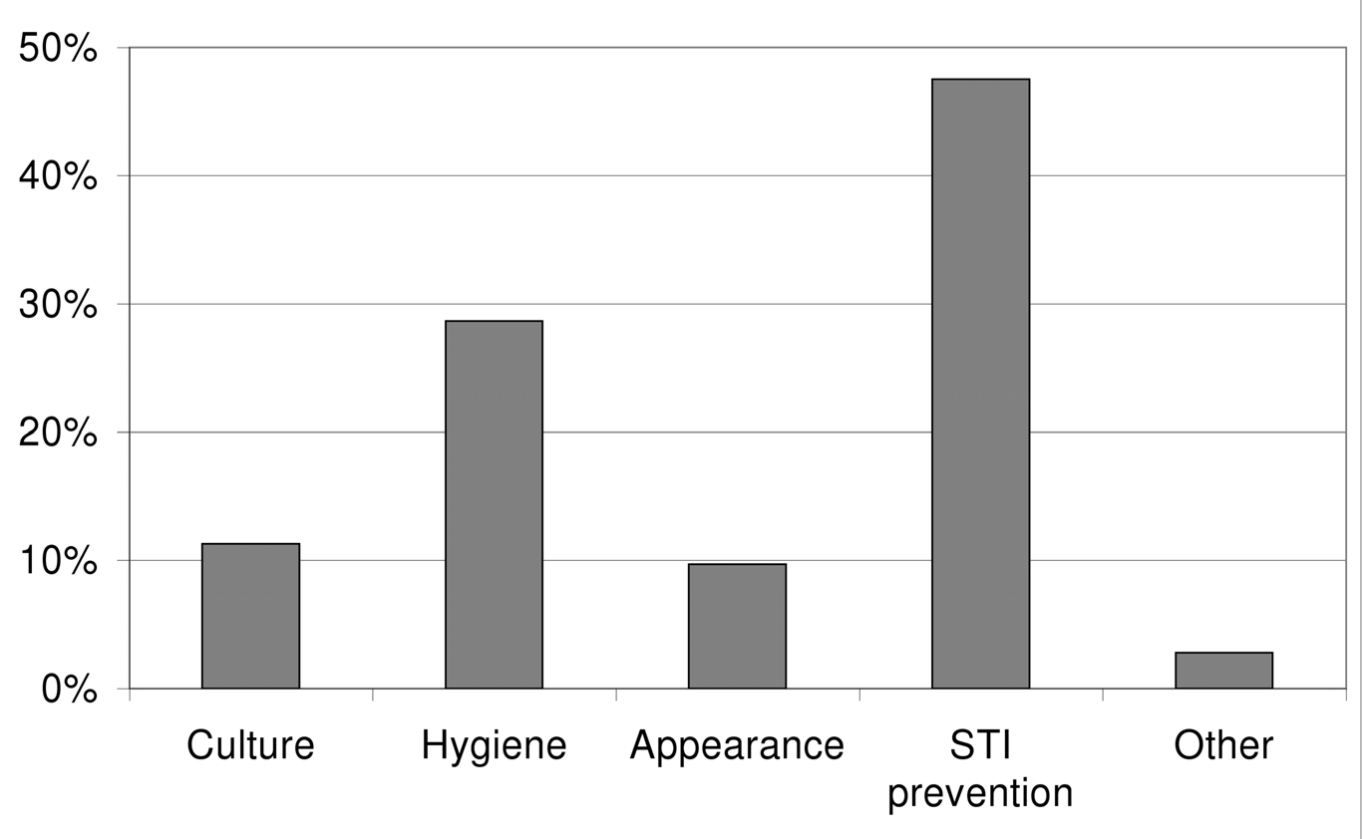
Reasons for getting circumcised.

On the basis of the postsurgery questionnaires, and as indicated in Figure 3, 39% of participants reported that friends and/or family were the communication sources that had influenced them to join the project. The remaining 61% cited direct project outreach and communication activities, such as household visits, street talks, pamphlets, radio, seeing the project's main center, and clinic talks. Among these direct communication sources, household visits and street talks were identified as most influential (30% and 27% of direct sources, respectively). Figure 4 illustrates the reported reasons for undergoing AMC, which were reduction in the risk of STI acquisition for 48% of participants, and better hygiene for 29%. Only 11% reported undergoing AMC because of their culture. Furthermore, over 94% of participants found that the information provided during the group session and the individual counseling was either good or very good. On-site wait time for counseling was considered short or acceptable for 75% of the participants, and 84% found on-site wait time for surgery acceptable. Over 84% of participants reported that they were either likely or very likely to adhere to the 6-wk postsurgery period of abstinence, and only 6% stated that they were either unlikely or very unlikely to adhere to the recommendation. Lastly, the vast majority of participants (92%) were satisfied with the services provided by the medical circumcisers.

## Discussion

As indicated by the substantial levels of community support, AMC uptake, and participants' satisfaction, the roll-out of free medicalized AMC as an intervention against HIV in the Orange Farm community has been a success. The study has shown that the scale-up of comprehensive, high-quality AMC services can be achieved according to international recommendations and operational guidelines in a low-income African setting with low AMC rates and high HIV prevalence.

Three main factors have contributed to this success: community involvement, the outreach and communication strategies, and the quality of the services offered to participants.

Community involvement has been actively sought and, from the beginning of the project, political, traditional, religious, medical, and other community key stakeholders have been engaged and consulted. The CAB has been active and influential in garnering support for the project and generating helpful suggestions. Through these activities and the organization of community workshops, institutional support has been secured, partnerships with key community groups have been formed, and advocacy has been fostered. In addition, extensive qualitative and quantitative research was conducted in preparing the project to inform the community, and, more importantly, to assess and take into account its needs and expectations. All these activities have enabled the building of strong ties with the Orange Farm community, and the development of a sense of community ownership of the project, which ensured its acceptability and feasibility. In addition, the research team was already familiar with the Orange Farm setting and was known within the community since it had led the randomized clinical trial of the effect of AMC on HIV acquisition [1]. This connection probably facilitated the rapid implementation of the project.

Diverse communication strategies delivered collectively and at the individual level by trained fieldworkers and counselors have been used to increase project awareness, AMC and HIV knowledge, and to effectively drive demand for services, as reported by a large majority of participants. Outreach activities have been particularly important to make the project known since the National South African Campaign on Medical Male Circumcision has not been launched yet [28].

The project's team, mostly locally recruited, was highly motivated, experienced, and friendly. Every effort was made to provide prompt, high-quality services and to accommodate the participants' needs. There were no wait lists for services, Saturday appointments were offered, and the operating hours were flexible. Participants who had missed their day appointment could still come at a later date and undergo circumcision on that day. The surgical team operated until all scheduled and unscheduled surgery appointments had been honored. As a result, most participants reported high satisfaction with the services they received. This earned the project a good reputation and facilitated the intervention despite some resistance and protests from local groups who, at the beginning, were not convinced of the project' scientific relevance or its acceptability in the community.

Through the project, a sizeable proportion of eligible Orange Farm male residents have undergone AMC in less than 2 y, and uptake is still increasing. Participants have been from both traditionally circumcising and noncircumcising communities. Most reported that health concerns were more important than cultural factors when deciding to undergo AMC. This viewpoint is in agreement with findings from a review of male circumcision acceptability in sub-Saharan Africa, which found that AMC appeared to have become more an issue of personal choice than ethnic identity [17]. However, for young men who might prefer to undergo a traditional ritual, the project's collaboration with traditional circumcisers in providing safe AMC alongside traditional rites may be a way to limit the high rate of complications associated with traditional circumcision [29],[30]. Undeniably, the uptake will be further facilitated when messages in favor of AMC are delivered at a national level and by traditional leaders. In this regard, the recent call made in December 2009, by the Zulu King, for the revival of the practice of male circumcision to help fight the spread of HIV, is expected to increase AMC uptake among young Zulu men in the future. This practice had been abolished by Zulu leaders at the beginning of the 19th century [31]. Interestingly, in his statement, the King has recommended that medical doctors be involved.

The Bophelo Pele project has been meticulous in the organization of the surgery room, the training of medical staff, the establishment of task-sharing, the formulation of detailed standard operating procedures, and the standardization of the surgical technique used, in order to ensure safety and quality as well as to optimize costs, time, staff, and space. The project's surgery, which is suitable for low-income settings, can deliver a high volume of AMCs while adhering to international guidelines and recommendations. Results from internal and external audits show that the project offers quality services by international standards. In addition, as recommended by UNAIDS/WHO [5], the provision of AMC services has been successfully delivered as part of a comprehensive HIV prevention package. Indeed, the project has been a unique opportunity to test participants for HIV, educate them on broader HIV prevention strategies, and engage them on other aspects of their sexual health.

The Bophelo Pele project nonetheless has two main potential limitations. The first is that this study was carried out in the community where the first AMC trial was conducted, which could have had an influence on the study findings. However, three arguments suggest that this influence may be negligible: (a) the AMC trial ended in 2005, over 2 y before the implementation of the project, (b) the trial was conducted among a sample of about 3,000 men, who represent a small proportion of the total Orange Farm population, so it is unlikely that a large proportion of men from the community would have been informed of the trial through its participants, and, more importantly, (c) in the baseline cross-sectional survey, no evidence of such an influence was found, since only a small proportion of the surveyed men could correctly recall the trial findings. The second potential limitation concerns the financial and human resources allocated to the outreach and communication activities, which may seem intensive and not easily replicable in other low-income settings. However, once there is political engagement and government leadership is established, such intensive community outreach and communication activities may not be necessary.

This study identified three general challenging issues associated with AMC scale-up in Africa. Firstly, because AMC services are offered through the project regardless of HIV status for ethical reasons and to avoid stigmatization [5], the low uptake of VCT is of concern, particularly in light of the potential consequences of newly circumcised seropositive men resuming sexual activity prematurely. However in Kenya, where a large AMC project is being conducted, VCT acceptance was 22.8% before the introduction of provider-initiated testing and counseling [32]. In effect, this finding is in agreement with other studies conducted in South Africa, where uptake of HIV testing remains comparatively low [33]–[35], although provider-initiated VCT uptake is slightly higher [36]. A nationwide campaign encouraging citizens to undergo HIV tests was launched recently and VCT uptake is expected to increase in the foreseeable future. Secondly, the unsatisfactory proportion of participants coming of their own volition for their follow-up visit, despite our efforts, is also of concern. Operational research is needed to identify its causes and achieve improvements. Lastly, although research conducted in the context of this study has shown that AMC information recall was satisfactory after 4 mo, uncertainties remain about the long-term recall of counseling messages.

In the Bophelo Pele project, the organization of AMC service delivery is essentially vertical, aside from its association with local medical doctors, so it is not integrated into an existing health infrastructure. This organization has allowed for quick project implementation, but it has been argued that it could limit the adoption and dissemination of a health intervention [37]. Despite its vertical character, the project has forged strong collaborations with local NGOs, local public health care facilities, and private medical practices, and has put in place efficient referral systems for AMC, HIV, and STI care and management. The project has been designed as a catch-up intervention to generate a high number of AMCs in the shortest time among men aged 15 y and older, because it is expected to be most cost-effective and have the largest and most immediate impact on HIV incidence [7],[19]. In fact, the project is only meant to be sustained in the short term, until saturation level among adults has been reached. Once this is achieved, the provision of circumcision to children and newborns will be the next public health priority because it is more likely to be sustainable [38], although its cost-effectiveness and impact on the epidemic can only be evidenced after a few decades [19].

The generalization of AMC in South Africa is a foremost priority because the country contributes the largest proportion of uncircumcised adult men living in Southern and Eastern Africa, and a successful roll-out would fulfil over a quarter of the estimated AMCs that are needed in these regions [9]. The Bophelo Pele project was the first AMC community project to be implemented in Africa following the 2007 UNAIDS/WHO scale-up recommendations [5]. This project was set up at a site representative of South African communities in order to ensure that it could be replicated in other communities. A modeling study [9] has estimated that to generalize AMC in South Africa, an average of 549 (95% confidence interval [CI] 401–798) medical circumcisers, each performing ten AMCs a day for 5 y, would be required. This number could be overestimated since it is well below the current daily yield of the Bophelo Pele project's medical circumcisers, as shown in this study.

The Bophelo Pele project is well known internationally and has been visited by national and international public health officials and stakeholders. The project is already considered a model for the roll-out of comprehensive AMC services by numerous health and developmental agencies such as the Bill & Melinda Gates Foundation, Population Services International (PSI), The United States Agency for International Development (USAID), the U.S. President's Emergency Plan for AIDS Relief (PEPFAR), and by WHO and UNAIDS, which have used the experience gained from this project to inform the UNAIDS/WHO operational guidelines and recommendations to generalize AMC, known as MC MOVE (Model for Optimizing the Volume and Efficiency of Male Circumcision Services). Indeed, key characteristics of the project, such as the community mobilization strategies, its organizational structure, the innovative surgical organization, and the standardized surgical procedure, which this study has proven to be effective and adapted to low-resource settings, are compatible with and adaptable to a variety of health systems. To date, the project has already been successfully replicated at three sites in Zimbabwe, Swaziland, and Botswana.

Along with similar ongoing interventions in Southern and Eastern Africa, such as those conducted in Kenya and Uganda, the Bophelo Pele experience shows promise that AMC is a prevention method that can be scaled-up in the general population to fight the HIV epidemic in these regions. Now that the feasibility of AMC scale-up has been demonstrated, further research can be envisaged to precisely determine its impact on sexual behavior, and the extent to which it can curb the spread of HIV and other STIs at the population level.

## Supporting Information

Table S1 Quality of AMC messaging and information recall among Orange Farm Residents.
(0.03 MB PDF)

## Abbreviations

AMC: adult male circumcision
CAB: community advisory board
STI: sexually transmitted infection
UNAIDS: Joint United Nations Programme on AIDS
VCT: voluntary counseling and testing
WHO: World Health Organization
