# Supplementary material for: A Model for the Roll-Out of Comprehensive Adult Male Circumcision Services in African Low-Income Settings of High HIV Incidence: The ANRS 12126 Bophelo Pele Project
Source: PLoS Med. 2010 Jul 20;7(7):e1000309. doi: 10.1371/journal.pmed.1000309 (PMC2907271; doi:10.1371/journal.pmed.1000309)
Supplement: Table S1 — Quality of AMC messaging and information recall among Orange Farm Residents. (0.03 MB PDF) [file pmed.1000309.s001.pdf]

Table 1. Quality of adult male circumcision (AMC) messaging and information recall among Orange Farm Residents[a].

Women	Uncircumcised men	Circumcised men
Opinion about quality of documents prepared for the Bophelo Pele Project [b]		
- Clear, simple and straightforward	- Clear and easy to understand<br>- Wording used appropriate<br>- Appreciated that the documents were in local languages	- Clear and easy to understand<br>- Wording used appropriate
Information recall on AMC four months after document provision: **AMC and HIV risk**		
- All participants recalled that AMC reduced men's risk of HIV acquisition<br>- All participants recalled that AMC was not fully protective against HIV acquisition<br>- Most participants remembered that the protective effect was 60%	- All participants recalled that AMC was not fully protective against HIV acquisition<br>- Most participants recalled that AMC had a 60% protective effect<br>- Two participants thought that AMC had no effect on HIV acquisition	- All but one participant recalled that AMC had a protective effect on HIV acquisition<br>- Two participants thought that AMC was fully protective against HIV<br>- Other participants recalled that AMC was not fully protective against HIV and that the protective effect was above 50%
Information recall on AMC four months after document provision: **AMC and women**		
- All but two participants recalled that AMC did not protect women from HIV acquisition and that women received no direct benefits from AMC<br>- All participants agreed that a woman could still get infected with HIV by an infected sexual partner even if he was circumcised	- All participants recalled that AMC did not protect women from HIV acquisition and that circumcised men needed to practice safe sex<br>- All participants agreed that an HIV-positive man could infect his female partners even if he was circumcised	- All but one participant recalled that AMC did not protect women from HIV acquisition and that women received no direct benefits from AMC<br>- Men stated that it was important to discuss AMC with their female partners since they could incorrectly perceive themselves as protected
Information recall on AMC four months after document provision: **Abstinence period**		
- Most participants recalled that the abstinence period lasted six weeks<br>- All participants recalled that there should be no sexual activity for at least six weeks after AMC surgery	- All participants agreed that AMC healing would be completed after six weeks, although some thought that healing was complete when there was no pain and open wounds had closed<br>- All participants recalled there should be no sexual activity for at least six weeks after AMC surgery. All participants recalled that having sex during the abstinence period could delay healing, even if a condom was used	- Some participants reported that AMC healing took about two to three weeks depending on pain and wound healing<br>- All participants agreed that there should be no sexual activity for at least six weeks after AMC surgery
Information recall on AMC four months after document provision: **Sexual behavior** [c]		
- All participants recalled that men needed to reduce their risky sexual behavior even if they were circumcised to reduce their risk of HIV acquisition<br>- All but one participant recalled that consistent condom use was necessary even with circumcised sexual partners	- All but six participants recalled that risky sexual behavior could counteract the protective effect of AMC	- All participants recalled that circumcised men were still at risk of getting HIV<br>- All participants recalled that circumcised men still needed to use condoms<br>- Some men did not agree that risky sexual behavior could reduce the effect of AMC

[a] Among six focus group discussions, two included 38 women, two 40 uncircumcised men and two 36 circumcised men, aged 18 to 29.
[b] Pamphlets entitled "*What men should know about male circumcision*" (821 words) and "*What women should know about male circumcision*" (618 words)
[c] Condom use and number of sexual partners
